# Biologically Active and Antimicrobial Peptides from Plants

**DOI:** 10.1155/2015/102129

**Published:** 2015-03-01

**Authors:** Carlos E. Salas, Jesus A. Badillo-Corona, Guadalupe Ramírez-Sotelo, Carmen Oliver-Salvador

**Affiliations:** ^1^Departamento de Bioquímica, Universidade Federal de Minas Gerais, 31270-901 Belo Horizonte, Brazil; ^2^Unidad Profesional Interdisciplinaria de Biotecnología, Instituto Politécnico Nacional, Avenida Acueducto S/N, Colonia Barrio La Laguna Ticomán, 07320 Mexico City, Mexico

## Abstract

Bioactive peptides are part of an innate response elicited by most living forms. In plants, they are produced ubiquitously in roots, seeds, flowers, stems, and leaves, highlighting their physiological importance. While most of the bioactive peptides produced in plants possess microbicide properties, there is evidence that they are also involved in cellular signaling. Structurally, there is an overall similarity when comparing them with those derived from animal or insect sources. The biological action of bioactive peptides initiates with the binding to the target membrane followed in most cases by membrane permeabilization and rupture. Here we present an overview of what is currently known about bioactive peptides from plants, focusing on their antimicrobial activity and their role in the plant signaling network and offering perspectives on their potential application.

## 1. Introduction

No doubt proteins were designed to be versatile molecules. The number of functions in which they participate during metabolism supports this affirmation. Proteins act as defense, integrating the immunological system, as part of the enzymatic network required during metabolism, as a nutrient, as storage, contractile, structural, and motile molecules, as transporters, and as signaling and regulatory mediators. These are well-established functions for which proteins have gained undisputed roles. Aside from these functions other roles are associated with these molecules, such as antifreezers, sweeteners, and antioxidants. A relatively new role involves their ability to interact with cellular membranes in a nonreceptor-ligand type of binding.

Antimicrobial peptides (AMPs) are often the first line of defense against invading pathogens and play an important role in innate immunity [1]. The list of identified antimicrobial peptides has been growing steadily over the past twenty years. Initially, the skin of frogs and lymph from insects were shown to contain antimicrobial peptides, but now over 1500 antimicrobial peptides have been described, in living organisms including those from microorganisms, insects, amphibians, plants, and mammals [2].

In 1963, Zeya and Spitznagel described a group of basic proteins in leukocyte lysosomes endowed with antibacterial activity [3]. Later, Hultmark et al. [4] purified three inducible bactericidal proteins from hemolymph of immunized pupae of* Hyalophora cecropia*. The vaccinated insects survived a posterior challenge with high doses of the infecting bacteria, indicating the relevance of the bactericidal proteins. Additional research identified a 35-residue peptide (cecropin) as responsible for the antibacterial effect. Further investigation by Boman and other groups confirmed that antimicrobial peptides (AMPs) are distributed ubiquitously in all invertebrates investigated, generating academic and commercial interest [1, 5–9].

Because the rapid increase in drug-resistant infections poses a challenge to conventional antimicrobial therapies, there is a need for alternative microbicides to control infectious diseases [2, 10–13]. Bioactive peptides can fulfill this role because they display antibacterial, antiviral, antifungal, and/or antiparasitic activities. A comparative analysis of these molecules reveals that there are no unique structural requirements useful to discriminate these activities and to facilitate their classification. Most bioactive peptides have a high content of cysteine or glycine residues; the disulphide bridges that may be formed between cysteinyl residues increase their stability. Most of them contain charged amino acids, primarily cationic, and also hydrophobic domains. Both, *β*-sheets or *α*-helices, looped or extended, structures or combinations of these domains can be found in natural bioactive peptides [3, 6, 7, 14–24]; their length varies between 12 and 55 residues. There is evidence that cationic charged peptides are relevant for antibacterial or antiviral activity but few exemptions of anionic peptides also exist.

This review updates information on plant bioactive peptides. When little or no available information exists on a specific group, we use examples taken from other lifer forms, assuming that upcoming studies may reveal information on peptides whose attributes have not yet been found in plants. The review does not cover in detail the antimicrobial mechanism underlying the effect of bioactive peptides since two recent reviews on the subject were published [4, 5, 11, 14, 15, 25–31].

## 2. Antimicrobial Peptides Isolated from Plants

As mentioned above, AMPs are part of important immunological barriers to counter microorganism microbial infections and represent another aspect of the resistance phenomenon known as the hypersensitive response (HR). This phenomenon was described by H. Marshall Ward in cultures of leaf rust (*Puccinia dispersar* or* Puccinia triticina*) and by several plant pathologists 100 years ago [1, 5, 7, 8]. The hypersensitive reaction (HR) is considered the maximum expression of plant resistance to pathogen attack and is defined as a fast death of the plant cells associated with growth restriction and pathogen isolation. Cell death that happens during HR is considered a lysosomal-type of programmed cell death (PCD) or autophagy [2, 10, 12], unlike mammalian apoptosis. Also, signaling by resistance gene products (RGP) triggered during the HR response is not associated with death effectors (mammalian caspases), or with the death complex equivalent to the mammalian apoptosome. It is hypothesized that RGP signaling is required to initiate deployment of non-HR defenses, most likely via the production of so-called “dead signals” like ROS (reactive oxygen species), NO (nitric oxide), and SA (salicylic acid), all of them initiators of resistance in the absence of a HR [3, 14, 16]. Therefore, HR is viewed as part of a continuum of effects mediated by defense elicitors [4, 5, 15, 25, 27–29].

Although many AMPs are generically active against various kinds of infectious agents, they are generally classified as antibacterial, fungicides, antiviral, and antiparasitic. The antibacterial activity of peptides results from the amphiphilic character and presence of motifs with high density of positively charged residues within their structure [6–9]. This type of arrangement facilitates peptide attachment and insertion into the bacterial membrane to create transmembrane pores resulting in membrane permeabilization. The amphipathic nature of antimicrobial peptides is required for this process, as hydrophobic motifs directly interact with lipid components of the membrane, while hydrophilic cationic groups interact with phospholipid groups also found in the membrane.

The antifungal activity of AMP was initially attributed to either fungal cell lysis or interference with fungal cell wall synthesis. A comparison of plants antifungal peptides suggests a particular structural-activity arrangement involving polar and neutral amino acids [11–13, 32]. However, like for antibacterial peptides, there are no obvious conserved structural domains clearly associated with antifungal activity. The cell wall component “chitin” has been implied as fungal target for bioactive peptides [6, 7, 15, 17–24]. Peptide binding induces fungal membrane permeabilization and/or pore formation [4, 11, 14, 15, 26, 29–31].

The antiviral effect of some AMPs depends on their interaction with the membrane by electrostatic association with negative charges of glycosaminoglycans facilitating binding of AMP and competing with viruses [11]. Such is the case of the mammalian cationic peptide lactoferrin that prevents binding of herpes simplex virus (HSV) by binding to heparan moieties and blocking virus-cell interactions [3, 32–34]. Alternatively, defensins (described below) bind to viral glycoproteins making HSV unable to bind to the surface of host cells [25, 27]. The antiviral effect of peptides can also be explained by obstruction of viral interaction with specific cellular receptors, as shown during binding of HSV and the putative B5 cell surface membrane protein displaying a heptad repeat alpha-helix fragment. The effect was demonstrated with the synthetic 30-mer peptide that has the same sequence found in the heptad repeat that inhibits HSV infection of B5-expressing porcine cells and human HEp-2 cells [7, 15, 19, 20, 22–24]. Another mechanism involves the interaction between AMP and viral glycoprotein as shown with a retrocyclin-2 analogue that binds with high affinity (Kd = 13.3 nM) to immobilized HSV-2 glycoprotein B (gB2) while it does not bind to enzymatically deglycosylated gB2 [25, 28]. A less specific interaction between AMP and viruses causes disruption or destabilization of viral envelope yielding viruses unable to infect host cells [15, 17, 19, 21–24]. Finally, a peptide mediated activation of intracellular targets induces an antiviral effect as demonstrated with the antiviral peptide NP-1 from rabbit neutrophils that crosses the cell membrane migrating into the cytoplasm and organelles, followed by inhibition of viral gene expression in the infected cell. The proposed mechanism involves downregulation of VP16 viral protein entry into the nucleus that prevents expression of early viral genes required to propagate viral infection [4, 11, 26, 30, 31].

The initial characterization of molecules displaying AMP activity was followed by isolation of purothionin, the first plant-derived AMP. Purothionin is active against* Pseudomonas solanacearum*,* Xanthomonas phaseoli* and* X. campestris*,* Erwinia amylovora*,* Corynebacterium flaccumfaciens*,* C. michiganense*,* C. poinsettiae*,* C. sepedonicum,* and* C. fascians* [25]. Since then, several plant peptides have been discovered. The major groups include thionins (types I–V), defensins, cyclotides, 2S albumin-like proteins, and lipid transfer proteins [15, 19, 22–24]. Other less common AMPs include knottin-peptides, impatiens, puroindolines, vicilin-like, glycine-rich, shepherins, snakins, and heveins (Table 1) [35–44].

Full isolation of plant AMP has been attained in some cases. It is the case of lunatusin a peptide with molecular mass of 7 kDa purified from Chinese lima bean (*Phaseolus lunatus* L.) (Table 1). Lunatusin exerted antibacterial action on* Bacillus megaterium*,* Bacillus subtilis*,* Proteus vulgaris,* and* Mycobacterium phlei*. The peptide also displays antifungal activity towards* Fusarium oxysporum*,* Mycosphaerella arachidicola,* and* Botrytis cinerea*. Interestingly, the antifungal activity was retained after incubation with trypsin [45].

Another peptide, named vulgarinin, from seeds of haricot beans (*Phaseolus vulgaris*), with a molecular mass of 7 kDa showed antibacterial action against* Mycobacterium phlei*,* Bacillus megaterium*,* B. subtilis,* and* Proteus vulgaris* and antifungal activity against* Fusarium oxysporum*,* Mycosphaerella arachidicola*,* Physalospora piricola,* and* Botrytis cinerea*. Its antifungal activity was also retained after incubation with trypsin. Another example is a peptide from* Amaranthus hypochondriacus* seeds that displays antifungal activity (Table 1) [46, 47].

Both lunatusin and vulgarinin inhibited HIV-1 reverse transcriptase and inhibited translation in a cell-free rabbit reticulocyte lysate system, suggesting a similarity of action between these two peptides and that antimicrobial activity might be linked to protein synthesis [46]. Lunatusin also elicited a mitogenic response in mouse splenocytes [45] and proliferation of breast cancer MCF-7b cell line while vulgarinin inhibited proliferation of leukemia L1210 and M1 cell lines and breast cancer MCF-7 cell line [46].

A peptide named hispidulin was purified from seeds of the medicinal plant* Benincasa hispida* that belongs to the Cucurbitaceae family (Table 1). Hispidulin exhibits a molecular mass of 5.7 kDa, is composed of 49 amino acid residues, and displays broad and potent inhibitory effects against various human bacterial and fungal pathogens [48]. Two additional antifungal peptides with novel N-terminal sequences, designated* cicerin* and* arietin*, were isolated from seeds of chickpea (*Cicer arietinum*), respectively. These peptides exhibited molecular masses of approximately 8.2 and 5.6 kDa, respectively. Arietin expressed higher translation-inhibitory activity in a rabbit reticulocyte lysate system and higher antifungal potency toward* Mycosphaerella arachidicola*,* Fusarium oxysporum,* and* Botrytis cinerea* than cicerin. Both lack mitogenic and anti-HIV-1 reverse transcriptase activities [2, 49, 50].

There are also some studies on AMP peptides from dry seeds of* Phaseolus vulgaris* cv. brown kidney beans; these AMPs exhibit antifungal and antibacterial activity [2, 50, 51]. Another AMP (So-D1-7) was isolated from a crude cell wall preparation from spinach leaves (*Spinacia oleracea* cv. Matador) and was active against Gram-positive (*Clavibacter michiganensis*) and Gram-negative (*Ralstonia solanacearum*) bacterial pathogens, as well as against fungi, such as,* Fusarium culmorum, F. solani*,* Bipolaris maydis*, and* Colletotrichum lagenarium* [44].

Antiparasitic peptides are another group of bioactive peptides. Following an initial report describing the lethal effect of* magainin* isolated from Xenopus skin on* Paramecium caudatum*, another peptide (cathelicidin) confirmed the antiparasitic activity of AMPs [52–56].

Anthelmintic activity is also a recognized feature attributed to vegetable proteinases (Table 1). For instance, bromelain, the stem enzyme of* Ananas comosus* (Bromeliaceae), shows anthelmintic effect against* Haemonchus contortus* [52, 53], similar to the reference drug pyrantel tartrate. A similar effect was confirmed with proteinases from papaya (*Carica papaya*), pineapple (*A. comosus*), fig (*Ficus carica*), and Egyptian milkweed (*Asclepia sinaica*)* in vitro* against the rodent gastrointestinal nematode* Heligmosomoides polygyrus* [57]. The anthelmintic effect cannot be fully explained by the proteolytic effect of these enzymes, as the inhibited enzymes partially preserve antiparasitic activity. It is suggested that selected domains within the proteinase molecule different from the active site could be responsible for the antiparasitic effect (unpublished observations). The notion that specific regions within a protein are responsible for the biocide effect is supported by the observation that some AMPs become functional upon protein hydrolysis, like in egg [58, 59] and milk proteins hydrolysates [58, 60–63]. At present, there are not many studies on plant protein hydrolysates with antibiotic properties; this situation encourages the search in protein databases for motifs featuring the signature of AMPs.

Plant proteinases also display antifungal activity as demonstrated with latex proteinases from* Calotropis procera*,* Carica candamarcensis,* and* Cryptostegia grandiflora* [27, 60, 61]. Using a collection composed of* Colletotrichum gloeosporioides*,* Fusarium oxysporum*,* F. solani*,* Rhizoctonia solani*,* Neurospora *sp., and* Aspergillus niger*, fungal germination, growth, and IC_50_ were determined. The observed IC_50_ for* Rhizoctonia solani* with proteinases from* C. procera* was 20.7 ± 1.6 *µ*g/mL while with proteinases from* C. candamarcensis* was 25.3 ± 2.4 *µ*g/mL. Chitinases are also chitinolytic enzymes found in different plants that display antifungal activity [64].

*Plant Defensins*. There is no consensus about the size of defensins. According to some authors defensins are AMPs that range from 18 to 48 amino acids, while other groups define them as having 12–54 residues. Regardless of their size they contain several conserved cysteinyl residues structuring disulphide bridges that contribute to their stability. Two kinds of defensins have been described, *α*-defensin and *β*-defensin, the latter probably emerged earlier based on its similarity with insect forms. Defensins are among the best-characterized cysteine-rich AMPs in plants [27, 65]. All known members of this family have four disulphide bridges and are folded into a globular structure that includes three L-strands and a K-helix [65, 66]. Initially, these proteins were described in human neutrophils [66, 67], more specifically in granules of phagocytes and intestinal Paneth cells [67–71]. Later, they were described in human, chimpanzee, rat, mouse, marine arthropods, plants, and fungi [68–71].

Defensins are structurally classified in four categories, which correlate with morphological and/or developmental changes in fungi following treatment with defensins [72–75]. Defensins of group I cause inhibition of Gram-positive bacteria and fungi, and fungal inhibition occurs with marked morphological distortions of hyphae (branching); those of group II are active against fungi, without inducing hyphal branching, and are inactive against bacteria; those of group III are active against Gram-positive and Gram-negative bacteria but are inactive against fungi; while group IV are active against Gram-positive and Gram-negative bacteria, and against fungi, without causing hyphal branching. The selective action assigned to these four groups of defensins suggests that specific determinants within each group are responsible for targeting different groups of infectious agents.

Several defensins have been purified from plants. The PvD1 defensin from* Phaseolus vulgaris* (cv. Perola) seeds is a 6 kDa peptide (Table 1). Its N-terminal has been sequenced and the comparative analysis in databases shows high similarity with sequences of different defensins isolated from other plants species. PvD1 has been shown to inhibit the growth of yeasts,* Candida albicans*,* C. parapsilosis*,* C. tropicalis*,* C. guilliermondii*,* Kluyveromyces marxiannus,* and* Saccharomyces cerevisiae*. PvD1 also inhibits phytopathogenic fungi including* Fusarium oxysporum*,* F. solani*,* F. lateritium,* and* Rhizoctonia solani *[51, 72]. Analysis of cloned PvD1 cDNA yielded a fragment that contains 314 bp, encoding a 47-amino-acid polypeptide displaying strong similarity with plant defensins from* Vigna unguiculata* (93%),* Cicer arietinum* (95%), and* Pachyrhizus erosus* (87%).

An antifungal peptide with a defensin-like sequence and exhibiting a molecular mass of (7.3 kDa) was purified from dried seeds of* Phaseolus vulgaris* “cloud bean” (Table 1). The peptide exerted antifungal activity against* Mycosphaerella arachidicola* with an IC_50_ value of 1.8 *μ*M and it was also active against* Fusarium oxysporum* with an IC_50_ value of 2.2 *μ*M [52]. From lentil (*Lens culinaris*), a 47-amino-acid-residue (Lc-def) defensin was purified from germinated seeds (Table 1). The molecular mass (5.4 kDa) and the complete amino acid sequence were determined. Lc-def has eight cysteines forming four disulphide bonds; it shows high sequence homology with defensins from legumes and exhibits activity against* Aspergillus niger* [50, 76].

A 5.4 kDa antifungal peptide, with an N-terminal sequence highly similar to defensins and with inhibitory activity against* Mycosphaerella arachidicola* (IC_50_ = 3 *μ*M),* Setosphaeria turcica,* and* Bipolaris maydis*, was isolated from the seeds of* Phaseolus vulgaris* cv. brown kidney bean (Table 1). The antifungal activity of the peptide against* M. arachidicola* was stable in a wide pH range (3–12) and progressively decreases at pHs <2 and >12. Similarly, its activity remains stable between 0 and 80°C and partially declines between 90 and 100°C. Deposition of Congo red at the hyphal tips of* M. arachidicola* was induced by this peptide indicating inhibition of hypha growth. The lack of antiproliferative activity of brown kidney bean antifungal peptide toward tumor cells, in contrast to the presence of such activity seen in other antifungal AMPs, suggests that different domains are responsible for the antifungal and antiproliferative activities [50].

The biotechnological potential of defensins became evident following experiments aimed at increasing plant resistance to pathogens by genetic transformation of various recipient plants. In a number of cases increased resistance to specific pathogens was obtained in transgenic plants overexpressing a defensing gene [24].

## 3. Peptides from Plant Hydrolysates

Plant protein hydrolysates represent an option for production of bioactive peptides. Hydrolysis can be done enzymatically or under acidic conditions; the former is preferred because it is milder and effectively produces bioactive peptides from a variety of sources, like legumes, rice, chia seeds, and so forth. Particularly, studies with enzymatic hydrolysates from leguminous plants, like common bean (*P. vulgaris *L.), are relevant since this is a fundamental ingredient of human diet in several cultures and because it represents up to 10% of total proteins ingested in developing countries [77, 78].

The characterization of bioactive peptides released by hydrolysis demonstrates that they preserve their nutritional value, and at least, some of them behave as biologically active substances. Protein hydrolysates show antioxidant, antitumoral, antithrombotic, antimicrobial, or antihypertensive activities, thus qualifying as functional foods [77, 79]. Particularly, total hydrolysates (TH) or peptide fractions from leguminous such as chickpea, soya bean, pea, lentil, mung bean, and common beans demonstrate important antioxidant and angiotensin-I converting enzyme activities (ACE) [79, 80].

Our studies using concentrates following enzymatic hydrolysates from three common bean varieties of* P. vulgaris* L., plus black (PB), azufrado higuera (AH), and pinto saltillo (PS), show evidence of antimicrobial activity. The bactericidal activity determined by growth inhibition demonstrated that ten out of twelve bacterial strains were inhibited by these THs and also by the 3–10 kDa peptide fraction obtained by subsequent ultrafiltration of TH. The ultrafiltrate fraction from TH with cutoff of 1 kDa (<1 kDa) also demonstrated antimicrobial activity against* Shigella dysenteriae* in each of the bean varieties (PB, AH, and PS) at 0.1, 0.4, and 0.3 mg/mL, respectively [81]. A similar antimicrobial activity was seen in beans* Phaseolus lunatus* digested with pepsin followed by pancreatin [81]. Both TH and the partially purified peptide fraction (<10 kDa) exhibited antimicrobial activity against* Staphylococcus aureus *and* Shigella flexneri*. The largest antimicrobial effect was seen with the <10 kDa fraction and the determined MIC was 0.39 mg/mL against* S. aureus* and 0.99 mg/mL for* S. flexneri* [81].

Antiretroviral activity has also been described in alcalase hydrolysates of rapeseed (*Brassica napus*) protein. The antiviral effect seen in human immunodeficiency virus (HIV) is due to inhibition of the viral protease, possibly by a 6 kDa peptide. When rapeseed hydrolysate was purified by size-exclusion chromatography, two fractions of 6 kDa enriched in this protease inhibitor were isolated [82].

## 4. Role of Peptides in Plant Signalling

Since plants are stationary attached to earth, they must withstand aggressions from predatory activities by herbivores including man or pathogens and environmental variations like water supply, temperature changes, and manmade aggressions. To successfully meet these challenges, they have developed an efficient signaling network to elicit appropriate cellular responses. As in mammals, their signaling processes rely on efficient and specific interactions between organic molecules or simple ions (ligand) and their receptors to communicate and respond to these signals.

As result many plant peptides and proteins evolved as signaling molecules and play a key role in homeostasis, defense, growth, differentiation, and senescence. Most of these actions require the coaction of hormones (auxin, ethylene, abscisic acid (ABA), gibberellic acid, and cytokinins), acting as coregulators in these processes. As part of their defense strategies, a group of peptides evolved to inactivate microorganisms menacing plant essential functions. The antimicrobial peptides comprising this category are discussed in the previous section.

In this section, we focus on peptides whose main established functions provide a physiological attribute to the plant, but it should be noted that a peptide might participate in a defense strategy against infectious agents, while being at the same time a component of a metabolic function of the host plant without intervention of an infective agent. Some examples that illustrate this situation include a defensive peptide of 7.45 kDa from white cloud beans (*Phaseolus vulgaris *cv.) that shows reverse transcriptase inhibitory activity when probed* in vitro* [83, 84]. This type of effect does not follow a logical evolutionary explanation, unless a retroviral form yet unidentified is found in plants. In another similar situation, it is being shown that purothionin, the AMP from wheat endosperm, can substitute for thioredoxin/from spinach chloroplasts in the dithiothreitol-linked activation of chloroplast fructose-1,6 bisphosphatase, suggesting a role for the thiol carrier during regulation of redox molecules [83, 85].

Human *β*-defensins also display diverse immune related functions in addition to their antimicrobial activity. Such is the case of human *β*-defensin-2 that promotes histamine release and prostaglandin D2 production in mast cells. The immune modulatory role of *β*-defensin-2 has been further studied following the finding that *β*-defensin-2 binds to the chemokine receptor CCR-6, the cognate receptor for macrophage inflammatory protein-3*α*/CCL20 [85, 86]. Secretion of protein-3*α* along with other cytokines is linked to migration of immature dendritic cells from blood to the skin and from sites of inflammation to local lymph nodes triggering activation of memory specific T cells [86, 87]. In addition, *β*-defensins are associated with stimulation of toll-like receptor-4, thus serving as an additional mechanism for amplification of the innate host defense response [87, 88]. In summary, it is evident that at least some antimicrobial molecules evolved from host metabolites and share other functions.

In plants, most of these signaling molecules are found in seeds, highlighting the necessity to preserve the genetic material that represents the informational basis to sustain the species. Following* in silico* screening in* A. thaliana* about 15 peptide families were identified plus additional groups described in other species, most of them monocot [88, 89]. Aside from partial repositories available like in the case of secreted peptides in* A. thaliana* obtained by* in silico* analysis of unannotated sequences [89, 90], PhytAMP, a database dedicated to antimicrobial plant peptides http://phytamp.pfba-lab-tun.org/main.php [90, 91], C-PAmP, a database of computationally predicted plant antimicrobial peptides http://bioserver-2.bioacademy.gr/Bioserver/C-PAmP/ [2, 91], the antimicrobial peptide database that includes an algorithm to determine Boman's index http://aps.unmc.edu/AP/FAQ.php [2, 92] or attempts to identify a specific family of signaling peptides [88, 92], no comprehensive database is available that deposits all the signaling peptides described to date. The annotation of these sequences would be valuable to identify and catalogue new peptide sequences that continuously emerge.

Signaling peptides encompass a myriad of highly diversified sequences showing variation within and across species and without a common phylogenetic origin. These circumstances defy the efforts to classify them as a single group [88, 93–95]. A classification attempt involving their suggested functions includes homeostatic, innate immune responses (defensive), expansion and proliferation, organ maintenance and organogenesis, and sexual related functions. Three peptide classes, natriuretic class (PNP), phytosulfokines (PSK), and rapid alkalinization factors (RAF), participate in homeostatic functions. PNP has been purified from several species [93–96]. A number of effects are attributed to PNP, such as H^+^, K^+^, and Na^+^ fluxes in roots probably mediated by cGMP [96–98], transient increase of cGMP levels, water uptake in mesophyll cells, water exit from xylem, and osmotic dependent protoplast swelling [97–99]. Unconfirmed evidence suggests that a leucine-rich brassinosteroid receptor (AtBR1) displaying guanylyl cyclase activity and kinase-like structure could act as natriuretic peptide receptor [99, 100].

PSKs are sulfated pentapeptides containing two sulfated Tyr residues synthesized as precusrsors. The ligand acts on phytosulfokine receptors (PSKR) which are leucine-rich repeat receptors displaying guanylate cyclase activity [100, 101].

The alkalinization RALF factor and homologues (RALF-like) are 5 kDa peptides, expressed in a tissue specific manner. Its role in roots is associated with hair growth control by modulation of intra- and extracellular pH [101, 102]. Indirect effects such as K^+^ and Ca^+2^ currents are linked to proton-pump changes [102, 103]. Some of the actions attributed to RALF may involve the participation of abscisic acid too [103–105].

The meristematic region at the top of the shoot responds to many actions related to growth and differentiation of the plant. The apical meristem contains stem cells that generate signaling peptides following a genetic program influenced by the surrounding habitat. The CLE family includes several groups of peptides capable of triggering signaling pathways. CLV3 is a 13-residue peptide of this family that plays a fundamental role by promoting stem cell differentiation during meristematic development [104–106]. A battery of transgenic assays using the recombinant forms of CLE peptides showed that overexpression of 10 CLE genes, like the CLV3 positive control, resulted in growth arrest at the shoot apical meristem [106, 107]. Contrary to the initial observation that fully active CLV3 was 13 residues long, a recent report provides evidence that CLV3 must contain five additional N-terminal residues that are critical for optimal activity* in vitro* [107–110].

The identified receptor for CLV3 is CLV1 plus the isoforms CLV2 and CRN [108–112]. These leucine-rich repeat receptors are membrane associated and display cytoplasmic kinase domain. Additional genes include POL, KAPP, and WUS that likely act as downregulators of this pathway [111–113]. Senescence-controlling proteins have been also identified; BAX inhibitor-1, the evolutionarily conserved cell death suppressor found in yeast, is also present in plants. It seems that BAXI-1 acts by delaying methyl jasmonate-induced senescence [106, 113]. A similar situation is encountered at the other end (root meristem) where CLE peptides influence root growth, as well. Overexpression of CLE peptides following transformation assays was observed for CLV3, CLV9, CLV10, CLV11, and CLV13 and linked to root growth inhibition, while overexpression of CLE2, CLE4, CLE5, CLE6, CLE7, CLE18, CLE25, and CLE26 was associated with root growth induction [106, 114]. Overall, it seems that these CLE peptides keep a balance between differentiation and stem cell status.

Vascular meristematic development is controlled by a CLE bearing twelve-amino-acid peptide designated by Ito et al. [114, 115] as tracheary differentiation inhibitory factor (TDIF). The cognate receptor (TDR) contains a leucine-rich repeat and kinase domains as described earlier and is located at the membrane of procambial cells. Its putative role involves suppression of xylem vessel differentiation [115, 116].

The self-incompatibility response during fertilization of hermaphrodite plants is another example of signaling mechanism. In Brassicaceae the pollen determinant and ligand are the S-locus pollen peptide (SP11) [116–118]. The interaction between SP11 and the S-locus receptor kinase (SRK) triggers a signaling cascade leading to inhibition of self-pollination. Structural features of the ligand and the receptor play an important role in this interaction, in such way that interaction between noncognate pairs of ligand receptors fails to occur. Aside from SP-11, additional pollen factors might be needed for the appropriate interaction between SP11 and SRK receptor [118–120].

An additional signaling pathway involves the genesis of stomata pores on leaves that regulate gas exchange with the environment. In* A. thaliana*, such family of ligands designated as “epidermal patterning factor like” (EPFL) contain eleven members ranging in sizes between 5 and 9 kDa. While EPF1 and EPF2 inhibit stomata formation, EPFL9 stimulates stomata formation [119–121]. A recent report shows evidence that EPFL5 represses stomata development by inhibiting meristemoid maintenance in* A. thaliana* [121, 122]. The membrane receptors for transducing the EPFL signal are ER, ER1, and ER2 as described by Shpak et al. [122, 123]. Plant pores adjust their opening/closure condition in response to nutritional needs and humidity by changing turgor pressure of guard cells through intervention of CO_2_ and ABA leading to increase in Ca^+2^ sensitivity (for a review see [123, 124]). Also, the number of stomata cells varies as a function of CO_2_ via a light induced mechanism. A recent review discusses the various pathways involving stomata development in* A. thaliana* [124, 125].

## 5. Perspectives

Biologically active peptides represent an excellent example of the advantage of the evolutionary process capable of selecting assortments of amino acids with antimicrobial activity. In the likely event of evolutionary changes within the target offender, new forms of peptides naturally emerge to counter the resistant infectious agent. Changing the assortments of amino acids and/or their order in the peptide are simple alternatives that evolved successfully in living systems during millenniums. Research is needed to elucidate the strategies adopted by life forms producing AMPs to counter the defensive plots posed by invading germs.

Several options are available to improve the quality, selectivity, durability, and safety of AMPs. For instance, the functional and immunological properties of proteins can be improved by partial hydrolysis and the resulting hydrolysate can be used in food systems as additives for beverage and infant formulae, as food texture enhancer or as pharmaceutical ingredient [125, 126]. Bioactive peptides can be computationally modeled, genetically manipulated, and expressed in different systems to serve a practical purpose. In addition to their microbicide activities, other intriguing functions (opioid, antithrombotic, immunomodulatory, and antihypertensive) are emerging [58, 126, 127]. These attributes provide natural alternatives with potential to be used as food ingredients in a variety of applications [58, 127].

Another promising application of AMPs relates to their use on bacterial biofilms. Biofilms are thin layers of microorganisms that colonize onto surfaces, such as implants, dental plaques, ear skin, intestine, and occasioning highly challenging infections and diseases. Several studies demonstrate the efficacy of AMPs into blocking biofilm formation. Singh et al. [127, 128] showed that lactoferrin and LL-37, a human cathelicidin AMP or its derivative, blocked formation of* P. aeruginosa* biofilms at concentrations lower than those required to kill the planktonic cells and, also, reduced biofilm thickness of colonized* P. aeruginosa* by 60% and destroyed microcolony structures of treated biofilms. It also was found effective against both Gram-positive and Gram-negative bacteria [128, 129]. In addition, AMPs have potential to be used in treating persister cells, which are latent phenotypic variants highly tolerant to antibiotics [129, 130].

Since membrane integrity is essential for bacterial survival regardless of the metabolic stage of the cell and because AMPs target the membrane, they show good potential to kill persister microbes. In a recent study, a synthetic cationic peptide, (RW) NH2, was found to kill more than 99% of* E. coli* HM22 persister cells in planktonic culture [15, 19, 22–24, 130].

## Figures and Tables

**Table 1:** Selected plant antimicrobial peptides.

Peptide	Biological activity	Peptide size	Reference
Thionins (types I–V)	Antibacterial	45–47 residues	[15, 22–24]

Thionein: alpha-1-purothionin (*Triticum aestivum) *	Antibacterial	5 kDa 45 residues	[15, 25, 81]

Cyclotides: kalata B1 and B2 (*Oldenlandia affinis*)	Antibacterial, Antifungal, insecticide nematicide	28–37 residues	[15, 19, 22–24]

2S albumin-like *Malva parviflora*, *Raphanus sativus *	Antibacterial, allergen	105 residues	[15, 24]

Lipid transfer proteins (LTPs) (*Zea mays*)	Antibacterial	90–95 residues	[15, 22–24]

Knottin-peptides: PAFP-S (*Phytolacca americana*) knottin-type (*Mirabilis jalapa*)	Antibacterial	36-37 residues	[15, 35–43]

Puroindolines: PIN*A* and PIN*B* (*Triticum aestivum*)	Antibacterial	13 kDa	[15, 35–43]

Snakins (*Solanum tuberosum*)	Antibacterial	63 residues, 6.9 kDa	[15, 35–43]

Heveins (*Hevea brasiliensis*)	Antibacterial and antifungal	43 residues, 4.7 kDa	[15, 35–43]

Peptides (*Phaseolus vulgaris*)	Antibacterial and antifungal	2.2 and 6 kDa	[2, 49, 50]

Peptide PvD1 (*Phaseolus vulgaris*)	Antibacterial and antifungal	6 kDa	[60, 75]

Defensin-like (*Phaseolus vulgaris*)	Antibacterial	7.3 kDa	[15, 50]

Defensins (*Triticum aestivum* and *Hurdeum vulgare*)	Antibacterial and antifungal	5 kDa	[25, 53]

Lunatusin (*Phaseolus lunatus*)	Antibacterial^a^ and antiviral	7.0 kDa	[45]

Vulgarinin (*Phaseolus vulgaris*)	Antibacterial, antifungal, and antiviral	7.0 kDa	[46]

Hispidulin (*Benincasa hispida) *	Antibacterial and antifungal	5.7 kDa	[48]

Lc-def (*Lens culinaris*)	Antifungal	47 residues	[37, 79]

Cicerin (*Cicer arietinum*)	Antifungal and antiviral	8.2 kDa	[49, 60, 61]

Arietin (*Cicer arietinum*)	Antifungal and antiviral	5.6 kDa	[36, 49, 60, 61]

Peptide So-D1 (*Spinacia oleracea*)	Antifungal and antibacterial	22 residues	[36, 44]

Ay-AMP *Amaranthus hypochondriacus *	Antifungal	3.18 kDa	[47]

PR1, PR2 Chitinases (*Vitis vinifera*)	Antifungal	26 and 43 kDa	[19, 38, 41, 64]

Proteins from latex of *Calotropis procera *(CpLP)	Antifungal	13 kDa	[38, 60, 61]

Proteinases from *Carica candamarcensis*, *Carica papaya* and *Cryptostegia grandiflora* (Cg24-I)	Antifungal	23–25 kDa	[36, 60, 61]

Impatiens (*Impatiens balsamina*) Ib-AMP1, Ib-AMP2, Ib-AMP3, and Ib-AMP4	Antibacterial	20 residues	[36, 52, 53, 57]

Shepherins (*Capsella bursa-pastoris*)	Antibacterial and antifungal	28 residues	[38, 41]

Vicilin-like (*Macadamia integrifolia*)	Antibacterial and antifungal	45 residues	[38]

Peptides^a^ (*Brassica napus*)	Antiviral	ND	[82]

Proteinases from *Ananas comosus*, *Carica papaya, Ficus carica*, and *Asclepias sinaica *	Anthelmintic	23-24 kDa	[52, 53, 57]

^a^Mitogenic activity; ND: not determined.
